# COVID-19 and Alzheimer’s Disease: A Literature Review

**DOI:** 10.3390/medicina57111159

**Published:** 2021-10-25

**Authors:** Louis Hardan, Dimitar Filtchev, Ratiba Kassem, Rim Bourgi, Monika Lukomska-Szymanska, Hassan Tarhini, Fouad Salloum-Yared, Davide Mancino, Naji Kharouf, Youssef Haikel

**Affiliations:** 1 Department of Restorative Dentistry, School of Dentistry, Saint-Joseph University, Beirut 1107 2180, Lebanon; louis.hardan@usj.edu.lb (L.H.); rim.bourgi@net.usj.edu.lb (R.B.); 2 Department of Prosthetic Dental Medicine, Faculty of Dental Medicine, Medical University of Sofia, 1000 Sofia, Bulgaria; d.filchev@fdm.mu-sofia.bg; 3 Department of Biological Sciences, American University of Beirut, Beirut 1107 2020, Lebanon; rhk28@mail.aub.edu; 4 Department of General Dentistry, Medical University of Lodz, 251 Pomorska St., 92-213 Lodz, Poland; monika.lukomska-szymanska@umed.lodz.pl; 5 Department of Infectious and Tropical Diseases, Assistance Publique Hôpitaux de Paris, Bichat-Claude Bernard University Hospital, 75018 Paris, France; hassan.tarhini@aphp.fr; 6 Department of Medical Laboratory, The General Authority of the Syrian Arab Red Crescent Hospital, Damascus 0100, Syria; fouad.yar@gmail.com; 7 Department of Biomaterials and Bioengineering, INSERM UMR_S 1121, Biomaterials and Bioengineering, 67000 Strasbourg, France; endodontiefrancaise@outlook.com (D.M.); youssef.haikel@unistra.fr (Y.H.); 8 Department of Endodontics, Faculty of Dental Medicine, Strasbourg University, 67000 Strasbourg, France

**Keywords:** Alzheimer’s disease, coronavirus infections, COVID-19, pandemics, SARS-CoV-2

## Abstract

There are a number of potential implications for the field of Alzheimer’s disease (AD) stemming from the global spread of “SARS-COV-2”. Many studies that were conducted by Cleveland Clinic researchers identified a link between COVID-19 infection and brain abnormalities seen in people with AD. This article explains the association between COVID-19 and AD and how people with AD are affected by COVID-19, whether directly or indirectly. First, this article begins by explaining AD and its types, then giving an overview about COVID-19, its symptoms and the associated complications. Then, direct and indirect consequences of COVID-19 on people experiencing AD are discussed briefly. Some management strategies are recommended at the end of this article in addition to a future perspective on this topic. This article concludes by summarizing the main points mentioned about the association between COVID-19 and AD.

## 1. Introduction

The COVID-19 pandemic is causing global morbidity and mortality. Older people presented a five times higher risk of mortality than the general population, [1] straining health systems and disrupting societies, as well as putting individuals with AD and related dementias (ADRD) at risk of significant harm [2]. COVID-19 shows adverse impacts on the brain by reducing the level of consciousness [3]. Patients with AD are more vulnerable to infections with COVID-19 because they may not adequately recall or understand any of the suggested public health precautions (e.g., physical distancing and use of facemasks) [1,3]. In addition, physical distancing is not feasible for those who depend on others to perform their daily activities and attend to their basic needs, especially those with severe symptoms of dementia who experience major physical disability [4]. Moreover, older patients with dementia have a higher risk of being infected with COVID-19 and a high risk of mortality from COVID-19 infection. As a result, these patients have to experience longer lockdown periods to recover from the infection or to protect themselves from being infected [1,5]. Consequently, long lockdown periods contribute to more severe neuropsychiatric symptoms and worse behavioral disturbances as a direct result [5]. 

## 2. Overview on Alzheimer’s Disease

AD is a neurodegenerative illness that affects the brain and causes the loss of cognitive and neurological capacities over time [1]. 

The condition worsens over time. It gradually deteriorates the patient’s memory, thinking, and social skills, as well as his or her ability to carry on a simple conversation. Patients eventually lose their capacity to perform even the most basic daily chores [6]. 

Some of these issues can influence other physical processes of the body as Alzheimer’s progresses, such as swallowing, balance, and bladder control. Dehydration, malnutrition, and breathing food or fluids into the lungs are all major health issues that can lead to death. A person with AD is susceptible to pneumonia and other infections [7]. 

AD, which accounts for 60 percent to 80 percent of dementia cases in adults, is the most frequent cause of dementia [8,9]. 

Although most people get symptoms of AD in their mid-60 s, this disease is not considered a natural part of aging. The symptoms might affect people between the ages of 30 and 60 in certain uncommon circumstances. AD affects about 200,000 Americans under the age of 65, according to the Alzheimer’s Association [9]. 

After being diagnosed with AD, a person can live a normal life for four to eight years. Severe loss of brain function in advanced stages of AD can lead to a variety of difficulties for sufferers. However, patients can enjoy normal lives for up to twenty years depending on other conditions [10]. 

The causes of AD are still unknown due to a lack of reliable data. AD, on the other hand, is thought to be caused by a combination of biological malfunctions, deficits, heredity, lifestyle, and environmental variables that impact the brain over time, according to scientists [11]. 

### 2.1. Causes of Alzheimer’s Disease

Many factors play a significant role in the occurrence and development of AD [1,4,10]. It is crucial to identify the main causes of AD because recognizing these causes helps in finding solutions to mitigate its influence on the body. Some of the main causes are:

- Beta-amyloid plaque production: elevated cholesterol levels in the blood cause the creation of beta-amyloid plaques. When proteins cluster together, they form a hard, insoluble plaque. Plaque builds up between neurons in the brain, inhibiting cell communication and function. These cells eventually die as a result of this. When brain cells deteriorate and die, they lose their ability to process, store, and retrieve data. As a result, one of Alzheimer’s symptoms is memory loss [12]. AD is associated with hyperphosphorylation of tau protein and accumulation of amyloid-β peptide in the brain [13].
- Multi-pathogen infections: the interaction of several potential pathogens with neurodegeneration and neuroinflammation in AD has been reported [13,14]. Various pathogens, including viruses (Herpes simplex virus type 1) and oral infectious pathogens, especially periodontal infections caused by *Porphyromonas gingivalis*, a key pathogen in chronic periodontitis, was found in AD cases. However, other *bacteria*, such as *Helicobacter pylori*, which is associated with chronic gastric diseases, and *Chlamydophila pneumoniae*, which is implicated in chronic and lower-respiratory-tract diseases, also to play a role in AD [14].
- Neurofibrillary tangles: Tau proteins are disrupted by an increase in enzymes called tau kinases. They clump together and create neurofibrillary tangles as their structure changes. The tangles injure brain cells by disrupting cell communication [15].
- Blood flow deficiency: A lack of blood flow to the brain prevents the transfer all of the necessary nutrients to the cells. Blood clots, on the other hand, harm blood vessels. As a result, the memory-related areas of the brain, such as the amygdala and the hippocampus, do not receive enough blood, and this leads to the deterioration of the brain function [16].
- Inflammation: When the body is in peril, inflammatory indicators such as cytokines play a critical role in battling infection or injury. However, as inflammatory indicators in the body rise, cytokines begin attacking regions of the brain, causing damage to healthy neurons [17,18].

### 2.2. Types of AD

AD can be classified into two main types:

- Early-onset AD is a rare form of the disease, with only about 10% of people reported to be suffering from it. Early-onset Alzheimer’s symptoms emerge in people in their 30 s to mid-60 s. Genetic alterations passed down from parents to offspring are the most frequent cause [7,8,9,19].
- The most frequent type of AD is late-onset, which manifests symptoms in the mid-60 s. This type of AD is not produced by one gene only. One gene, however, could be a risk factor. The apolipoprotein E gene (APOE) has one allele (a variant form of a gene) that raises a person’s risk of developing this type of Alzheimer’s [19].

## 3. Overview on COVID-19 Disease 

Coronaviruses are RNA viruses that cause common respiratory infections in humans and they are an infectious agent responsible for mild respiratory tract infections [20]. The world has known two epidemics of fatal severe acute respiratory syndrome related to the betacoronavirus: severe acute respiratory syndrome coronavirus “SARS-COV-2” in 2002 [21] and Middle East respiratory syndrome coronavirus (MERS-COV) in 2012 [22].

Since December 2019, a third fatal Coronavirus “SARS-COV-2”, which caused severe bilateral pneumonia has spread throughout the world, starting in Wuhan, China [23]. This coronavirus has been known by “SARS-COV-2” and was similar to bat SARS-like-COVZXC21 and human “SARS-COV-2”. “SARS-COV-2” caused COVID-19. By October 2021, the World Health Organization (WHO) had noted more than 200,000,000 confirmed cases of COVID-19 infection and near 5,000,000 deaths due to this virus [23,24]. The primary mode of “SARS-COV-2” transmission is through direct contact via respiratory secretions (through coughs or sneezes, for example). Indirect transmission by contaminated surfaces or other specimens (blood, stool, etc.) is uncertain [24]. The Centers for Disease Control (CDC) indicates that patients with mild to moderate “SARS-COV-2” infection can transmit the virus for no longer than seven to 10 days from symptom onset, and 20 days for severe infections and immunocompromised and older patients [25]. 

The majority of patients infected with “SARS-COV-2” virus presented with mild to moderate respiratory symptoms, fever, cough, dyspnea, and fatigue. They recovered without need of any specific therapy [26]. Other symptoms include sore throat, anosmia, sputum production, and headache [27]. 

Elderly people and those with comorbidities such as cardiovascular disease, diabetes, chronic respiratory disease, AD, and cancer, on the other hand, are more likely to develop severe diseases requiring hospitalization and oxygen support [28]. 

COVID-19 infection may be complicated in severe cases by acute respiratory distress syndrome, septic shock, acute kidney injury, cardiac injury, and thromboembolic phenomenon. Many patients end up being transferred to intensive care units for monitoring, therapeutic anticoagulation, antibiotics, non-invasive ventilation support and intubation [29]. Older age, male sex, obesity and cardiac comorbidities were noted as risk factors for mortality [28]. 

Critically ill patients and older patients may present a cytokine storm after COVID-19, which is caused by the immune system’s dysregulation and overreaction to the virus. It can be more harmful because it increases inflammation [30]. 

Patients with COVID-19 infection present abnormal laboratory findings, which usually include decreased leucocytes, lymphopenia, eosinopenia, and increases C-reactive protein and ferritin. In addition, absolute lymphocyte count could be an indication for the prognosis of COVID-19 severity [31]. Thoracic CT scanners show bilateral ground glass opacities. 

Many efforts evaluate novel treatment of COVID-19 infections. Nevertheless, no anti-viral treatment has been validated to this point. Remdesivir shows a four day reduction in hospital stay without improved odds of survival. Treatment with hydroxychloroquine and azithromycine, lopinavir /ritonavir, and interferon shows no effect. 

Scientists have developed a COVID-19 vaccination using mRNA technology. It is now in production and is 95 percent effective against the virus [32]. 

## 4. The Impact of COVID-19 on Alzheimer’s Disease

### 4.1. Direct Neurological Consequences of COVID-19 on People with Alzheimer’s Disease

COVID-19 infection causes various neurological symptoms in three ways: the direct effects of “SARS-COV-2” on the nervous system, para infection, and neurological complications. COVID-19 may cause altered mental status, encephalopathy, neuro-cognitive syndrome (dementia-like), psychosis, cerebrovascular events, and others. 

“SARS-COV-2” enters respiratory cells via angiotensin converting enzyme-2 (ACE2) receptors and uses the transmembrane protease serine 2 (TMPRSS2) [33]. ACE2 is expressed by smooth muscle cells in brain vessels as by alveolar cells [34]. This can have a direct effect on central nervous system (CNS). The invasion of the CNS by “SARS-COV-2” is suggested by its analogy to another coronavirus [35]. 

Several potential paths of entry were proposed in the literature:

- “SARS-COV-2” enters the brain via the olfactory system. In fact, loss of smell is common in COVID-19 infection. MRI hypersignals of the olfactory epithelial cortex indicate infection especially with the presence of ACE2 receptors and TMPRSS2 in the olfactory epithelium [36]. The virus could enter by nerve terminals and spread into the brain as described by Dubé et al. [37]. Therefore, it is not evident that the virus reaches the olfactory neurons in this manner.
- Via blood-brain barrier (BBB): the virus reaches the brain by infecting the endothelial cells [35]. The presence of ACE2 receptors, other putative “SARS-COV-2” receptors, and inflammatory cytokines like interleukin (IL)-6, IL-1b, tumor necrosis factor (TNF), and IL-17 disrupt the BBB and may facilitate the entry of the virus to the brain endothelial cells [38]. The presence of pre-existing neurological disease or comorbidities increases the permeability of BBB.
- Infiltration with infection immune cells: infected immune cells can transfer virus to the brain. It is unclear if “SARS-COV-2” can enter immune cells for infection and transport to the CNS [39]. Chen et al. demonstrated the presence of “SARS-COV-2” nucleocapsid proteins in CD68 lymphocytes and macrophage; nonetheless, it is not clear whether it is the normal process of phagocytosis or infected cells [40]. In addition, many cerebral autopsies reveal a lack of immune cells [41].

Moreover, the COVID-19 pandemic worsens behavioral symptoms in uninfected AD patients. COVID-19 has been reported to influence cognitive functions and can possibly invade the brain, leading to cognitive dysfunction [42]. Patients with AD have been among the most affected in the early stages of the COVID-19 pandemic due to the direct effects and numerous indirect effects of the virus.

People with dementia are more vulnerable to contracting “SARS-COV-2” and spreading it because they may not be able to comprehend, carry out, or recall any of the recommended public health measures (e.g., physical distancing, use of face masks). Those who exhibit agitation, roaming, or disinhibition are more likely to catch and transmit the infection. For those who rely on others to complete their fundamental activities of daily living (e.g., bathing), such as those with more severe dementia with concurrent considerable physical handicap, physical separation is not an option [4]. 

Many dementia or elderly patients live in care facilities, and individuals in congregate living settings are generally in close quarters with one another and share common areas (e.g., dining and living rooms), putting them at risk of infection. Furthermore, because infected older people may present non-specific symptoms such as altered general activity, falls, or delirium rather than the typical COVID-19 symptoms of fever, cough, and difficulty breathing, their informal or professional caregivers may become infected if they do not take the necessary precautions [43]. 

Patients with dementia are more likely to have a high viral load because they are unable to comply with the health measures. A majority of these dementia patients may come from care homes with high rates of infection. Furthermore, if hospitals and intensive care units (ICUs) are overburdened, and ventilators or personal protective equipment (PPE) are in low supply, health-care rationing may be necessary, which could result in older individuals or those with dementia being denied intensive care [44].

“The respiratory syndromes of “SARS-COV-2” have got the most attention while neurological co-manifestations have received the least, though more than one-third of the patients had neurological symptoms”. All the neurological symptoms are reported during the initial stage of the infection. Inflammation-induced mediators have been observed in people infected with COVID-19. Many inflammatory cytokines are related to the status of AD progression and inversely correlated with immune responses [45]. Patients suffering from AD may forget common precautions for decreasing the risk of infection like washing their hands regularly, keeping a social distance, and wearing masks. The Center for Disease Control in the United States of America has suggested strict risk reduction measures for people with a history of dementia with specific recommendations, such as necessary reminders for regular, everyday hygiene practices, putting alarms in the bathrooms to remind them to wash their hands with soap for 20 s, and to wear a face mask in order to cover their nose and mouth [46].

Older people with dementia can develop a more serious infection from COVID-19 with more severe symptoms. Although precise estimates of mortality rates associated with AD do not yet exist, the access rate in emergency rooms, hospitalization, and mortality from infection with COVID-19 is higher in patients with AD than in healthy elderly people. In fact, it should be taken into consideration that advanced age and suffering from chronic medical conditions such as heart or lung disease or diabetes may increase the risk of severe progression and death by COVID-19 [47].

Interestingly, it was also suggested that a commonly used drug called memantine for AD might play a protective role against COVID-19 infection through inhibiting neurotoxicity and viral replication. This is due to the fact that memantine plays a role in preventing excess calcium in cells. This characteristic is very important in treating AD and has an antiviral potential [46]. COVID-19 can trigger the release of cytokines and can lead to the formation of a cytokine storm, with an increase in the levels of IL-1 and IL-6. Elevated levels of these cytokines can increase the widespread inflammation with damages to cellular mechanisms [48]. COVID-19 can stay inside some neurons without being toxic [49]. “The resulting cytokine storm in COVID-19 can cause a series of small punctate strokes without causing immediate neurological deficits” [49]. When these patients leave the hospital after being infected with COVID-19, they may experience poor attention and memory. Therefore, it is important for these patients to see a neurologist if they feel they still have cognitive issues.

**Figure 1:**
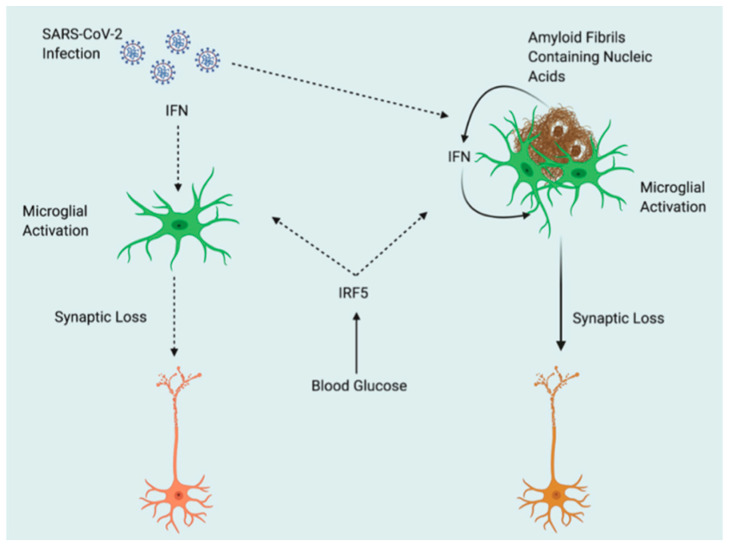
Potential Interactions Between “SARS-COV-2” and AD. Type 2 diabetes (T2D) acts as a predisposing factor for AD. Elevations in blood glucose resulting from T2D may exacerbate pathology in both AD and COVID-19, or comorbidity of the two, through increased activity of interferon regulatory factor 5 (IRF5). Type I interferons (IFN) mediate inflammation after viral infection and in response to nucleic acid containing amyloid fibrils, eventually leading to synaptic loss. Amyloid fibrils may entrap viral particles, leading to further enhancement of IFN response. Solid line arrows indicate proven mechanisms, dotted line arrows indicate theoretical mechanisms; from ref. [50] with authorization.

“SARS-COV-2” and AD: Possible Interactions Diabetes type 2 (T2D) is a risk factor for AD (Figure 1). 

Increased activity of interferon regulatory factor 5 (IRF5) may aggravate pathology in both AD and COVID-19, or the comorbidity of the two, as a result of blood glucose elevations caused by T2D. Inflammation is mediated by type I interferons (IFN) during viral infection and in reaction to nucleic acid-containing amyloid fibrils, which leads to synapse loss. Amyloid fibrils may entrap virus particles, enhancing the IFN response even further. The global spread of “SARS-COV-2” has a variety of potential ramifications for the study of AD. Neuroinflammation is a well-known characteristic of neurodegeneration and plays a key part in the pathogenesis of AD. COVID-19’s immune response and excessive inflammation may hasten the progression of brain inflammatory neurodegeneration, and elderly people are more vulnerable to severe “SARS-COV-2” infection outcomes. After “SARS-COV-2” infection, people with type 2 diabetes (T2D) are more likely to develop AD and have more severe results [50].

Dementia puts patients at a higher risk of infection. People with dementia and the elderly are all linked to severe disease and a higher mortality rate once infected. Reduced consciousness, delirium, stroke, and perhaps intracranial inflammation or viral neuro-invasion are all risks for older patients with more severe disease. Pre-existing brain vulnerabilities, such as a lack of brain resilience/cognitive reserve, may put dementia patients/older patients at greater risk of neurological problems, including irreversible cognitive decline [51]. 

### 4.2. Indirect Consequences of COVID-19 on People with Alzheimer’s Disease

During the coronavirus pandemic, people with AD are particularly at risk because those people have limited access to technological information available through the media and the internet. People with AD who live alone are at the greatest risk for rapid viral diffusion and unfavorable consequences [52]. A crucial factor to take into consideration in people with AD is to understand that the effects of the virus remain for a long period and require medical follow-up [53].

During the COVID-19 pandemic, a very important scientific discussion was undertaken as to whether to socially isolate people with AD or not in order to reduce the diffusion of the infection. A survey done in Italy in residential structures that involve AD patients showed that the mortality rate increased by 94% compared with the previous years before coronavirus struck. It was also revealed that in many small towns in southern Italy, the outbreaks were very scarce, while the presence of a senior residential structure for AD patients was the major factor resulting in turning the whole area into a red zone [54]. 

It has been reported that long lockdowns and confinement during the pandemic impacts the neuropsychiatric conditions in AD patients, especially those with very low cognitive function. The physical measures taken during the lockdown have particularly been present in patients with AD because social distancing increased loneliness and affected mental health among patients with AD, who were confined to their homes for months. These social and physical restrictions influenced their mental health, and patients with AD experienced more neuropsychiatric issues. These issues include anxiety, depression, and hallucinations. A positive correlation was reported between the duration of confinement and worsening of the symptoms of AD because the longer the confinement, the higher the level of distress [55].

Therefore, people with severe cognitive defects due to AD and dementias form one of the populations who are at the greatest risk of negative outcomes during the lockdown. The decrease in cognitive functions and the influence of pathology on the quality of life have serious effects on the family and on the person who assists the sick person at home. The need to stay close to patients suffering from AD and the need to take care of them were associated with a significant role in deteriorating quality of life and with an increased risk of disease and death. Therefore, the caregiver condition and the obligation to stay behind people with AD in order to help them and take care of them were associated with an increased risk of disease due to COVID-19 and an increase in the mortality rate due to COVID-19 [56].

Moreover, people with AD and advanced forms of dementias should take into consideration the drugs that are given to them daily and their possible effects on the course of COVID-19 infection. It is important to know that AD continues to progress even during the pandemic period and in the following phase. In addition, patients suffering from AD who were infected with COVID-19 should be particularly cared for and paid special attention to. They should not be left alone at home or any other place and it is very important to implement remote monitoring systems for all people suffering from chronic medical conditions and in particular those with AD [57]. 

## 5. Management Strategies 

Some management strategies can be taken into account and followed by elderly people with AD in order to protect themselves from COVID-19. 

Factors such as correct nutrition, including proper water and food intake, a sufficient amount of daily physical and cognitive exercise, and proper management of daily pharmacological therapies and medical devices must be taken into consideration, including the psychological well-being of frail. elderly people, [56,57].

It is also vital to consider the COVID-19 pandemic’s indirect consequences on Alzheimer’s sufferers. Because elderly people are more likely to die after contracting “SARS-COV-2”, it is crucial to isolate them and limit their contact with other people during the pandemic. Time spent with caregivers and family, as well as social engagement and daily activities, are all thought to assist older people in avoiding cognitive impairment. As a result, while seclusion is important, it may raise the risk of cognitive deterioration in the elderly. While the complete impacts of the COVID-19 pandemic remain unknown, many Alzheimer’s patients will surely be affected [50].

## 6. Future Perspective 

Given that the COVID-19 pandemic is a once-in-a-generation problem confronting aging societies around the world, emergency response techniques for community-dwelling dementia patients under lockdown may not exist and are urgently needed. Because of the potential catastrophic consequences connected with COVID-19, the goal is to keep them from becoming infected [58]. Despite the fact that several studies have documented cognitive impairment in COVID-19 patients, suggesting that COVID-19 may play a role in the development of AD, there is currently a lack of acceptable data to substantiate this association. The following research may be required to answer this question. 

For starters, short-term laboratory investigations can be carried out right away. The key AD pathological consequences of “SARS-COV-2” infection, for example, can be examined using various AD animal models, including cognitive functions, the accumulation rate of amyloid plaque and NFT, and activation of immune cells in the brain, based on which information can be gathered on whether COVID-19 increases the risk of AD in the general population.

More importantly, hPSC-derived brain organoid models provide an effective platform for simulating the condition of “SARS-COV-2” infection in the brain of AD patients. The impact of “SARS-COV-2” infection on the expression of AD risk genes and neuron degeneration may be assessed using AD brain organoids, providing useful evidence regarding the association of COVID-19 with AD.

Second, the COVID-19 patient samples, which include blood samples taken during hospitalization as well as post-mortem tissues, are a useful study resource. There have been few studies looking at AD-related pathological alterations in COVID-19 patients’ post-mortem tissues, particularly brain tissues, to date.

Third, establishing a long-term follow-up system for COVID-19 patients is critical. With such a large number of confirmed COVID-19 cases, substantial cohorts of COVID-19 patients, particularly among the elderly, can be formed. Many significant retrospective studies could be conducted after long-term follow-up to determine whether COVID-19 survivors have an elevated risk of AD.

## 7. Conclusions

“SARS-COV-2” has triggered a global COVID-19 pandemic. Apart from attacking the respiratory system, evidence suggests that “SARS-COV-2” can enter the CNS via hematogenous and neural routes, causing neurological problems such as cognitive impairment, neuro-inflammation, APP metabolism dysfunction, long-term hospitalization and delirium, post-COVID-19 syndrome, and other mechanisms.

On the other side, AD has been identified as one of COVID-19’s most common CNS comorbidities, putting a greater burden on patients, society, and the economy. COVID-19 morbidity and mortality are greatly increased in AD patients due to age, AD-induced direct and indirect pathologic alterations, drug-drug interactions, nutritional issues, and a lack of self-care and cognitive capacities. Furthermore, in the COVID-19 pandemic, seclusion or contact limitation has a deleterious impact on uninfected AD patients and inhibits AD prevention. As a result, all of these findings point to the need for better understanding of COVID-19’s underlying neurobiology, which will be explained in future research.

Future research on the link between COVID-19 and AD can take the following forms: (1) to determine how “SARS-COV-2” infects CNS cells and invades the CNS; (2) to investigate the causes of increased COVID-19 mortality in AD patients and develop treatment strategies; (3) to demonstrate the effects of COVID-19 on AD-relevant pathological and behavioral changes using animal models and patient samples; and (4) to create novel solutions that will allow patients to get the care they need despite COVID-19’s anticipated long-term impacts.

## Data Availability

The data that support the findings of this study are available from the corresponding author upon reasonable request.
